# Synthesis of silver nanoparticle-decorated hydroxyapatite nanocomposite with combined bioactivity and antibacterial properties

**DOI:** 10.1007/s10856-021-06590-y

**Published:** 2021-08-23

**Authors:** Soo-Ling Bee, Yazmin Bustami, A. Ul-Hamid, Keemi Lim, Z. A. Abdul Hamid

**Affiliations:** 1 School of Materials and Mineral Resources Engineering, Engineering Campus, Universiti Sains Malaysia, 14300 Nibong Tebal, Penang Malaysia; 2 School of Biological Sciences, Universiti Sains Malaysia, 11800 Pulau Pinang, Malaysia; 3 Center for Engineering Research, Research Institute, King Fahd University of Petroleum & Minerals, Dhahran, 31261 Saudi Arabia

## Abstract

Combination of bioactive material such as hydroxyapatite (HAp) with antibacterial agents would have great potential to be used as bone implant materials to avert possible bacterial infection that can lead to implant-associated diseases. The present study aimed to develop an antibacterial silver nanoparticle-decorated hydroxyapatite (HAp/AgNPs) nanocomposite using chemical reduction and thermal calcination approaches. In this work, natural HAp that was extracted from chicken bone wastes is used as support matrix for the deposition of silver nanoparticles (AgNPs) to produce HAp/AgNPs nanocomposite. XRD, FESEM-EDX, HRTEM, and XPS analyses confirmed that spherical AgNPs were successfully synthesized and deposited on the surface of HAp particles, and the amount of AgNPs adhered on the HAp surface increased with increasing AgNO_3_ concentration used. The synthesized HAp/AgNPs nanocomposites demonstrated strong antibacterial activity against *Staphylococcus aureus* bacteria, where the antibacterial efficiency is relied on the amount and size of deposited AgNPs. In addition, the in vitro bioactivity examination in Hank’s balanced salt solution showed that more apatite were grown on the surface of HAp/AgNPs nanocomposite when AgNO_3_ concentration used >1 wt.%. Such nanocomposite with enhanced bioactivity and antibacterial properties emerged as a promising biomaterial to be applied for dentistry and orthopedic implantology.

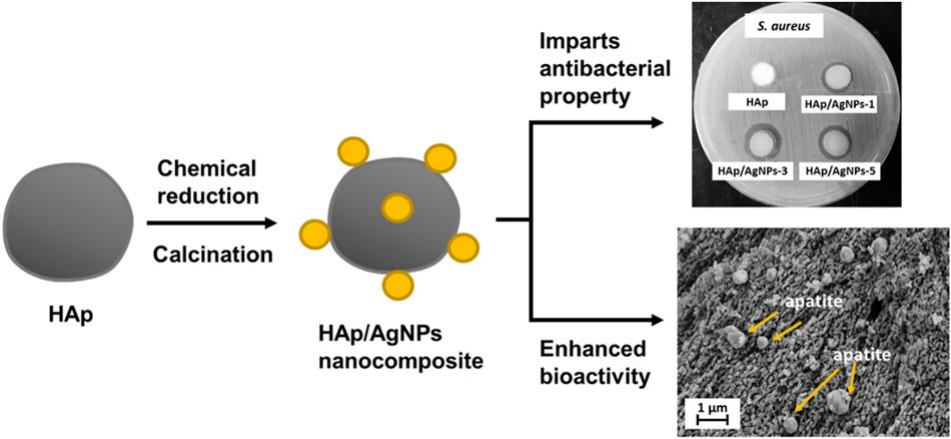

## Introduction

Hydroxyapatite (HAp, Ca_10_(PO_4_)_6_(OH)_2_), which belongs to the class of calcium orthophosphate, is a type of bioceramics that is chemically similar to the mineralized phase of natural bone and teeth. Owing to its intriguing biological properties such as bioactivity, biocompatibility, non-toxicity, osteoconductivity, and ability to provide direct chemical bonding with natural bone tissue, HAp has gained much prominence for both dental and orthopedic applications [1–4]. In dentistry, HAp has been widely used as bone grafts or implant materials to reconstruct bone defects caused by periodontitis [5, 6], as the scaffolds materials for periodontal regeneration [7, 8], as well as serves as the coating material for dental implants [9, 10].

Nevertheless, the use of HAp material for dentistry and bone implantology application can be impaired by implant-associated diseases such as peri-implant mucositis and peri-implantitis after surgical operations. This is because protein and other organic substances are readily adsorbed on HAp surfaces that make them susceptible to bacterial adhesion and colonization [11–13]. In order to circumvent this problem, antibiotics had been loaded into HAp formulation to prevent or cure implant-related infections by directly release antimicrobial agents to local region. However, due to the potential risk of increased antibiotic resistance that will affect the efficacy of antibiotic, the development of alternative antibacterial agents that possess a much lower tendency to cause bacterial resistance is on dire need [14].

Over the past decade, silver (Ag) compounds and silver nanoparticles (AgNPs) have garnered prominent consideration as effective antibacterial agents due to their broad spectrum of antibacterial properties against microorganisms, low bacterial resistance, and relatively low cytotoxicity toward mammalian cells [15–17]. Therefore, combination of Ag-based compound with HAp can be satisfactorily used as antibacterial implant material as it combines the bioactivity of HAp and bacteriostatic ability of Ag. However, direct blending of AgNPs with HAp together is not preferred as the small-size AgNPs tend to aggregate and thus will adversely debilitate its antimicrobial activity [18]. Doping of Ag^+^ ion into the HAp lattice structure is one of the most prevalently used approaches to impart antibacterial properties [19–24]. In a research conducted by Iqbal et al. [25], it was reported that the prepared Ag-doped HAp is active against common gram-positive (*Bacillus subtilis* and *Staphylococcus aureus*) and gram-negative (*Pseudomonas aeruginosa* and *Escherichia coli*) bacteria. Similarly, Ag-doped HAp as prepared by Chung et al. [26] exhibited microbial inhibitory property against *Streptococcus mutans*. Despite the significant contribution in rendering antibacterial properties, the structural instability and phase impurity issues as well as the complexity and difficulty in controlling the reaction parameters are among the drawbacks for preparing Ag-doped HAp materials [25, 26]. In a recent study, Hamouda et al. [27] proposed and demonstrated the success decoration of multi-walled carbon nanotubes (MWCNTs) with AgNPs onto the surface, in which the resulting nanocomposites exhibit broad antimicrobial spectrum against gram-positive and gram-negative bacteria. Based on the proposed synthetic route, the MWCNTs will serve as a substrate for the attachment of AgNPs via the carboxylic groups present on the MWCNTs surfaces. Likewise, it is reasoned that the decoration of AgNPs onto the HAp particle surfaces would be feasible, since HAp exhibits hydroxyl groups that can act as nucleating site for the attachment of AgNPs to avoid aggregation phenomena. Decoration of HAp with AgNPs could be a simple approach that offers benefit by imparting antibacterial properties without leading to the formation of other phase.

On the other hand, HAp can be synthesized chemically or extracted from natural food bio-wastes such as eggshell and bone wastes [28]. In fact, naturally derived HAp is more preferred over conventional synthetic HAp due to its chemical and structural resemblance to the natural bone that offers better biological properties to promote bone regeneration [29]. In our previous research, we reported the optimum parameter to extract natural HAp from chicken bone waste based on thermal calcination approach [30]. In the present work, we aimed to further prepare an antibacterial HAp/AgNPs nanocomposite via the chemical reduction and calcination methods. In this context, the natural HAp that was extracted from chicken bone bio-waste will serve as a solid support for the decoration and deposition of AgNPs to form HAp/AgNPs nanocomposite. The chemical reduction route employed was based on the use of *N,N-*dimethylformamide (DMF) and poly(vinyl acetate) (PVA) as reducing and stabilizing agents, respectively, to induce the formation and deposition of AgNPs on the HAp from Ag salt precursor.

As far as we are concerned, very little research conducted related to the decoration of HAp with AgNPs in enhancing antibacterial properties, especially using HAp derived from chicken bone waste. Furthermore, there is a scarce information reported regarding the Ag^+^ ion release in long-term studies. Indeed, the scrutiny of Ag^+^ ion release is crucial to ensure the released ion concentration is below the cytotoxic level over a sustain period of time. Herein, the physico-chemical, Ag^+^ ion release behavior, antibacterial and bioactivity properties of the resulting nanocomposites were investigated elaborately with respect to different Ag salt concentration used. Moreover, the formation mechanism of HAp/AgNPs nanocomposite was also proposed and elucidated in this study.

## Experimental procedure

### Materials

Chicken femur bone wastes were collected from several chicken rice shops in Nibong Tebal, Malaysia. PVA (Mw = 190,000) and silver nitrate (AgNO_3_) were purchased from BDH and Bendosen, respectively. DMF and sodium hydroxide (NaOH) were supplied from Merck. Mueller–Hinton agar (MHA) and Muller–Hilton broth (MHB) were procured from Oxoid. Sodium chloride (NaCl) and phosphate buffer saline (PBS) tablets were purchased from Sigma-Adrich. Hank’s balanced salt solution (HBSS, with calcium chloride and magnesium chloride) was supplied from Life Technologies Corporation. Throughout the experiment, distilled water was used to prepare aqueous solution. All chemicals were used without further purification.

### Preparation of HAp and HAp/AgNPs nanocomposite

HAp powder was extracted from chicken femur bone by thermal treatment according to our previous reported procedure [30] with slight modification. Briefly, the collected femur bone was washed, spongy bone was removed, and bone marrow was cleaned from the laterally sliced bone to obtain cortical bone. Next, the bone was boiled for degreasing, cleaned using NaOH (1 mol/L) and distilled water, and the dried cleaned bone was further grinded using an agate mortar and sieved (mesh size: 100 µm) into finer powder. In order to extract HAp, bone powder was further calcined using an electric furnace (Carbolite CWF 1100, Germany) under atmospheric condition at 600 °C with soaking time of 20 h in order to eliminate organic components.

Deposition of AgNPs onto the HAp surface was carried out based on chemical reduction of Ag^+^ ions, where DMF and PVA serve as reducing and stabilizing agents, respectively. Typically, AgNO_3_ with different concentration (i.e., 1, 3, and 5 wt.%) were dissolved in 5 mL of DMF under constant stirring for 3 h to form metallic Ag solution. Subsequently, 2.5 g of as-prepared HAp and 2.5 g of PVA were added into 50 mL of DMF and mechanically stirred for 1 h. Thereafter, the Ag solution was added dropwise into the mixture and allowed to stir overnight. It should be noted that all reaction was conducted under dark condition to minimize photo-activation of AgNO_3_. During the reduction reaction, the milky white slurry of HAp gradually transform into dark brown or gray suspension. After the completion of the reaction, the products were collected through filtration, washed with distilled water for several times, and dried in oven at 50 °C. Finally, the dried powders were calcined at 600 °C with soaking time of 1 h in order to remove the residual PVA. The prepared samples were denoted as HAp/AgNPs-*x* nanocomposite, where *x* refer to the concentration of AgNO_3_ added.

### Characterization techniques

#### X-ray diffraction

The phase compositions of all samples were determined by X-ray diffraction (XRD, Bruker Advanced X-ray Solution D8, Germany) using Cu-Kα radiation (*λ* = 0.15406 nm) over an angular range of 25–60°. X’Pert HighScore Plus software (The Netherland) was used for data analysis. The phase identification was confirmed by comparing the diffraction pattern of all samples with standard reference of HAp (JCPDS 09-0432) and metallic Ag (JCPDS 04-0783) available in the system software.

#### Fourier-transform infrared spectroscopy

Fourier-transform infrared (FTIR) spectroscopy was performed on the powder samples using a Perkin Elmer 2000 FTIR spectrometer (Massachusetts, USA). The FTIR spectrum of each sample was scanned over the range from 400 to 4000 cm^–1^ with 16 scans per specimen at a spectral resolution of 4 cm^–1^.

#### Field emission scanning electron microscopy–energy-dispersive spectroscopy

Field emission scanning electron microscopy (FESEM, Zeiss Supra 55VP, Germany) was utilized to investigate the surface morphology of the samples. Each sample was sputter coated with thin layer of platinum using sputter coating machine (Bio-Rad Polaron Division, SEM coating system) in order to make them conductive prior to analysis. The element contents of the HAp and all HAp/AgNPs samples were determined using energy-dispersive X-ray detector (EDX flash/60 detector from Bruker). The acceleration voltage during imaging and EDX analysis was 5.0 kV.

#### High-resolution transmission electron microscopy

Deposition of AgNPs on the HAp was evaluated using high-resolution transmission electron microscopy (HRTEM; JEOL JEM 2100F) using an accelerating voltage of 200 kV. Samples were prepared by ultrasonically dispersing the powders in ethanol before transferring a drop of the suspension onto the carbon grid. Gatan Digital Micrograph software was used to analyze the lattice distance of samples obtained from HRTEM micrograph.

#### X-ray photoelectron spectroscopy

X-ray photoelectron spectroscopy (XPS) was conducted to establish the elemental composition and the chemical state of Ag from the samples. The XPS wide and narrow scan spectra were acquired using X-ray photoelectron spectrometer (Kratos, AXIS Ultra DLD, UK) with an Al Kα X-ray (1486.6 eV) as excitation source under 4.8 × 10^–9^ torr ultra-vacuum environment inside the sample analysis chamber using the multichannel plate and delay line detector.

#### Ag^+^ ions release behavior

The release of Ag^+^ ions from the HAp/AgNPs nanocomposite samples was studied by soaking in PBS solution at 37 °C under constant shaking for 1, 3, 7, 14, 21, 28, and 42 days. After predetermined time intervals, the aliquot of the soaking medium was withdrawn, collected and immediately replenished by an equal volume of the PBS solution. The amount of Ag^+^ ion released by the sample at each soaking period was determined by inductively coupled plasma-optical emission spectrometer (ICP-OES, Perkin Elmer Optima 8000 ICP-OES spectrometer, USA).

#### Antibacterial study

The antibacterial activities of all the HAp/AgNPs nanocomposite samples were tested based on agar well diffusion method using *S. aureus* (ATCC 12600) as test microorganism. A single colony of tested bacteria was initially transferred into sterilized MHB and incubated at 37 °C for 24 h. Thereafter, the concentration of bacterial suspensions was adjusted using saline by comparing to the McFarland standard (0.5%, 10^6^–10^8^ CFU/mL) and daubed on the entire surface of MHA plates (contains wells of 10 mm in diameter). Subsequently, each sample suspension (400 mg/mL) was dispensed into respective wells and allowed to diffuse into the MHA prior to be incubated at 37 ± 2 °C for 24 h. After incubation, the zone of inhibition that surrounding each well was employed to access the antibacterial effect of the sample against the tested bacterial strain.

#### In vitro bioactivity study

In order to elucidate the effect of chemical reduction on the bioactivity property of HAp, all the prepared powder samples were immersed in HBSS at 37 °C and incubated up to 28 days. After predetermined incubation period, the soaked powder was removed by filtration and the filtered powders were rinsed with distilled water and air-dried before further characterized using FESEM.

## Results and discussion

### Visual observation

**Fig. 1:**
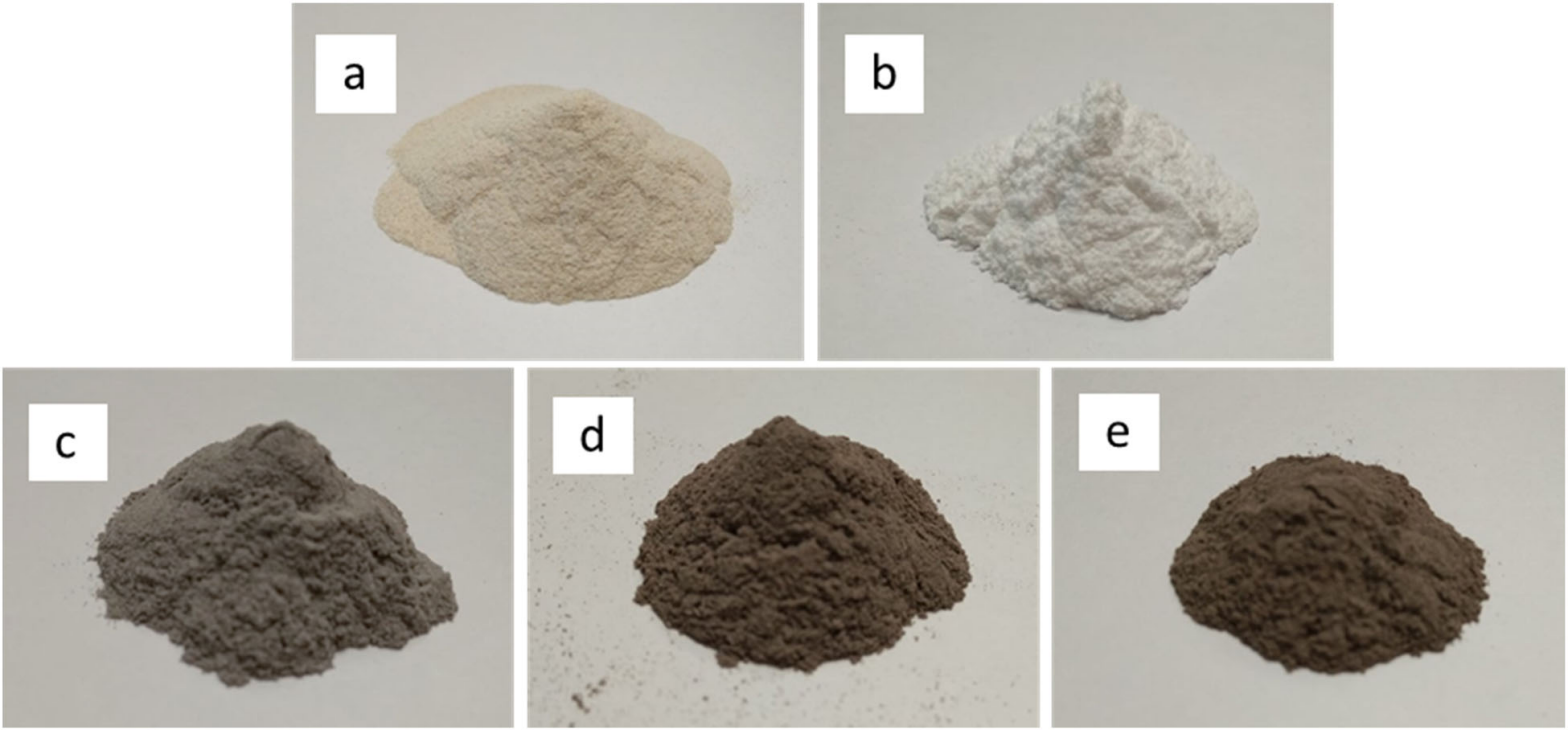
Photographic images of **a** un-calcined bone powder, **b** HAp powder, **c** HAp/AgNPs-1, **d** HAp/AgNPs-3, and **e** HAp/AgNPs-5 nanocomposites

Figure 1 shows the photographic images of un-calcined bone and prepared HAp powder as well as all the HAp/AgNPs nanocomposite samples obtained at different AgNO_3_ concentration. As can be seen, the color of chicken bone powder changed from light yellow to white upon calcination. This is owing to the combustion of organic constituents (such as fat and protein) from the bone sample during high thermal process, thereby leaving white color HAp phase behind. Similar to the research reported by Boainini et al. [31], the white HAp powder undergoes color change after chemical reduction and calcination process, which turned into light gray/grayish brown depending on the concentration of Ag salt used. This distinctive color change is due to the excitation of surface plasmon resonance that indicates the formation of AgNPs on the powder samples [32]. Furthermore, the color of the nanocomposite powders also becomes slightly darker upon increasing concentration of AgNO_3_ used. This can be ascribed to the formation of more AgNPs content in the resulting nanocomposites when a higher concentration of Ag salt precursor is used.

### XRD analysis

**Fig. 2:**
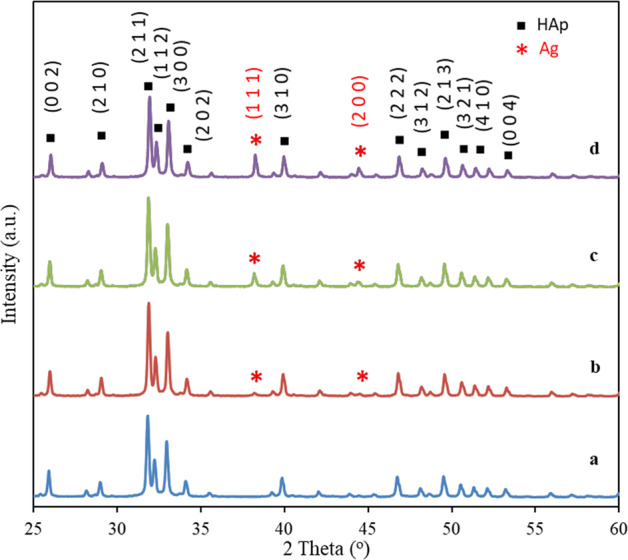
XRD patterns of **a** HAp, **b** HAp/AgNPs-1, **c** HAp/AgNPs-3, and **d** HAp/AgNPs-5 nanocomposites

Figure 2 shows the XRD patterns of the HAp and HAp/AgNPs nanocomposites formed with different AgNO_3_ concentration. The peaks were identified and indexed by comparing with the standard XRD pattern of HAp (JCPDS 09-0432) and metallic Ag (JCPDS 04-0783). As can be observed in Fig. 2a, the diffractogram of HAp shows the diffraction peaks at the 2*θ* values of 25.9°, 29.0°, 31.8°, 32.2°, 33.0°, 34.1°, 39.9°, 46.7°, 48.1°, 49.5°, 50.5°, 51.3°, and 53.2° that are assigned to the (0 0 2), (2 1 0), (2 1 1), (1 1 2), (3 0 0), (2 0 2), (3 1 0), (2 2 2), (3 1 2), (2 1 3), (3 2 1), (4 1 0), and (0 0 4) lattice planes of HAp, respectively [33]. For all HAp/AgNPs nanocomposite samples, additional peaks at ~38.2° and 44.4° that are corresponded to the (1 1 1) and (2 0 0) lattice plane of face-centered cubic (fcc) metallic Ag are also present along with other characteristic peaks of HAp [31]. These results are in accordance with previous reports by Rajendran et al. [34], which demonstrate the successful formation of HAp and HAp/AgNPs nanocomposites. Furthermore, no other peaks in addition to HAp and Ag are observed in all the HAp/AgNPs nanocomposites, indicating that the chemical reduction and calcination processes did not lead to the instability of HAp to form other phase. It is also apparent that the intensity of Ag characteristic peaks in the nanocomposite samples was gradually increased with the increased of AgNO_3_ concentration used during chemical reduction process. This dictate the increased amount of AgNPs formed in the nanocomposites when a higher AgNO_3_ concentration is used.

### FTIR analysis

**Fig. 3:**
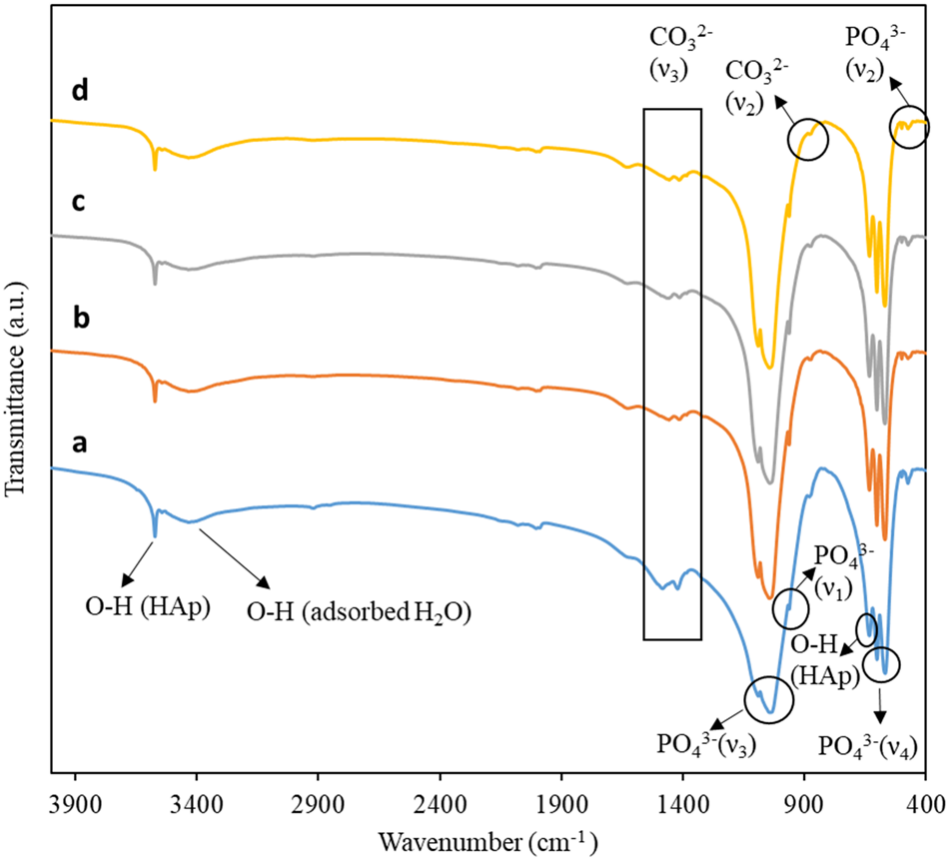
FTIR spectra of **a** HAp and **b** HAp/AgNPs-1, **c** HAp/AgNPs-3 and **d** HAp/AgNPs-5 nanocomposites

FTIR spectroscopy was employed to elucidate the functional groups present in all the synthesized samples. From the FTIR spectrum of HAp as depicted in Fig. 3a, the sharp peaks at 3572 and 633 cm^–1^ are corresponded to the O-H stretching and librational mode of structural hydroxyl group from HAp [35]. The characteristic bands at 1089–1043, 962, 601–567, and 500–423 cm^–1^ are assigned to the P-O asymmetric stretching (*ν*_3_), symmetric stretching (*ν*_1_), asymmetric bending (*ν*_4_), and symmetric bending (*ν*_2_) vibration of the phosphate group, respectively [36]. The broad peak at 3435 cm^–1^ is attributed to the O-H stretching of adsorbed H_2_O in the sample. The characteristic peaks at 1483–1422 and 880 cm^–1^, which are associated with the asymmetric stretching (*ν*_3_) and bending (*ν*_2_) vibration mode of CO_3_^2^^–^, were also detected in the FTIR spectrum of as-prepared HAp [37]. The presence of the adsorption bands associated with hydroxyl, phosphate, and carbonate vibrational peaks confirms the formation of carbonated HAp from calcination of chicken bone. All HAp/AgNPs nanocomposites, on the other hand, show characteristics band that are similar to pristine HAp without the formation of new peak. This result is in agreement with the XRD data that confirm the structural stability of HAp. By comparing to the Ag-doping approach as reported by previous literature [25] that can lead to the formation of additional phase (i.e., β-tricalcium phosphate), decoration of AgNPs onto HAp via chemical reduction and calcination as proposed in this study is advantageous for not causing the formation of other impurity phase. On the other hand, the characteristic band associated with *ν*_3_ PO_4_^3–^ has been shifted when relative to pristine HAp, which could be accreditated to the possible interaction between AgNPs with the phosphate group of HAp.

### FESEM-EDX analysis

**Fig. 4:**
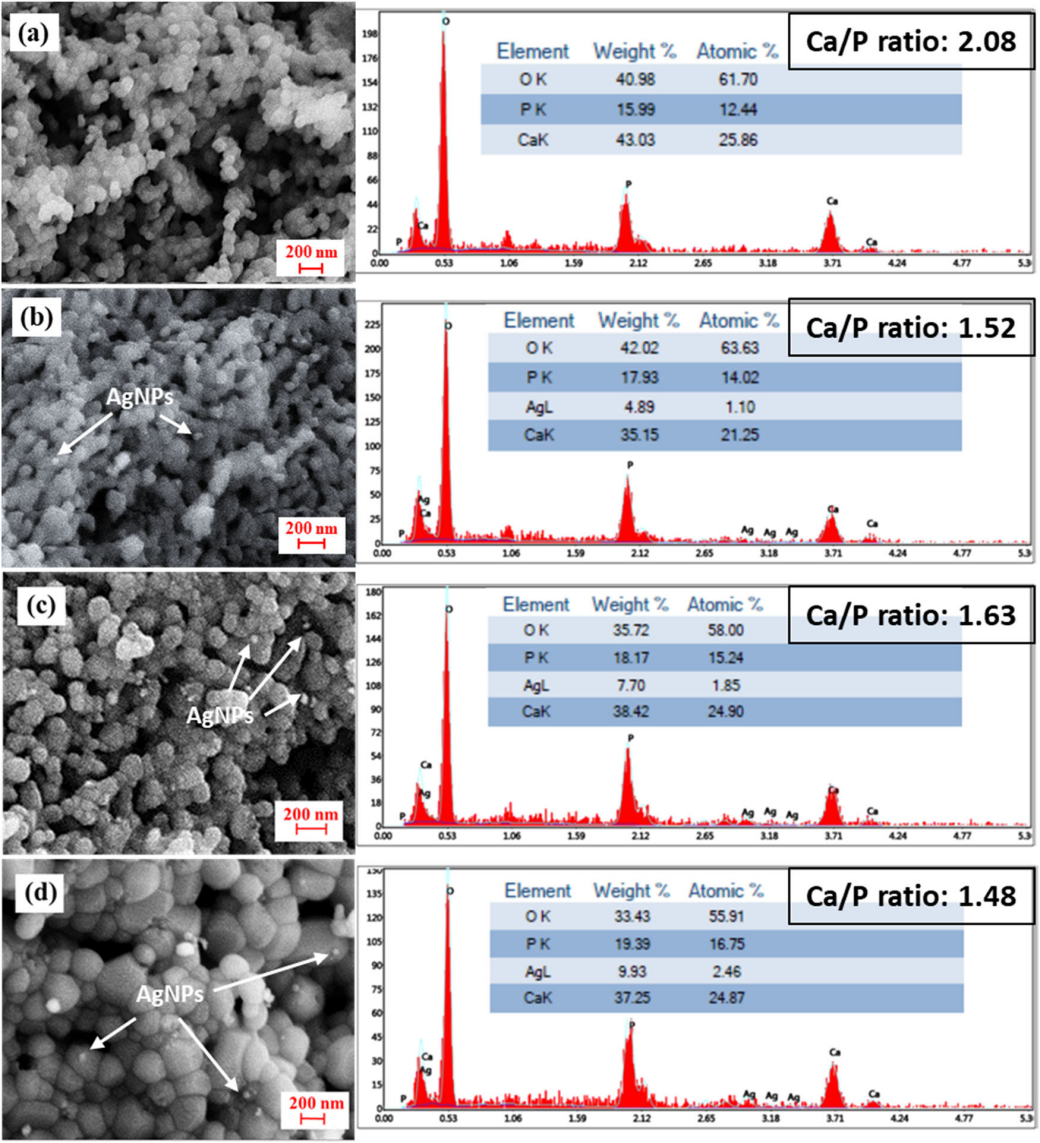
FESEM micrographs and EDX analyses of **a** HAp, **b** HAp/AgNPs-1, **c** HAp/AgNPs-3, and **d** HAp/AgNPs-5 nanocomposite powders

The surface morphologies and results of elemental composition of HAp and all HAp/AgNPs nanocomposite powder samples are depicted in Fig. 4. As can be seen from the FESEM micrographs, both calcined bone and nanocomposite samples showed an irregular grain-like morphology with interconnected porous network structure. As for the nanocomposite samples, it can be observed that some spherical AgNPs are decorated on the surface of HAp particles. Meanwhile, EDX analysis of all HAp/AgNPs nanocomposites shows that peaks related to oxygen, calcium, phosphorus, and silver elements thus further corroborate the co-existence of HAp and AgNPs in the powder samples. In agreement with the XRD result as beforehand mentioned, the weight percentage of silver in the nanocomposite samples increased proportionally with increasing AgNO_3_ concentration used. On the other hand, the Ca/P ratio of the samples obtained in the present work is all deviated from the stoichiometric HAp value of 1.67. The high Ca/P ratio obtained from calcined bone sample can be attributed to the presence of carbonate ions substituted phosphate ions in the HAp lattice structure, which is highly probable for HAp derived from biological source. This result is coherent to the Ca/P ratio value (2.01) of fish scale-derived HAp as reported in the past literature [38]. As for the nanocomposite samples, the decrement of Ca/P ratio value can be deciphered by the possible integration of Ag^+^ ions into HAp via cationic exchange of Ca^2+^ ions by Ag^+^ ions during chemical reduction process, thus leading to the formation of calcium-deficient HAp.

### HRTEM imaging

**Fig. 5:**
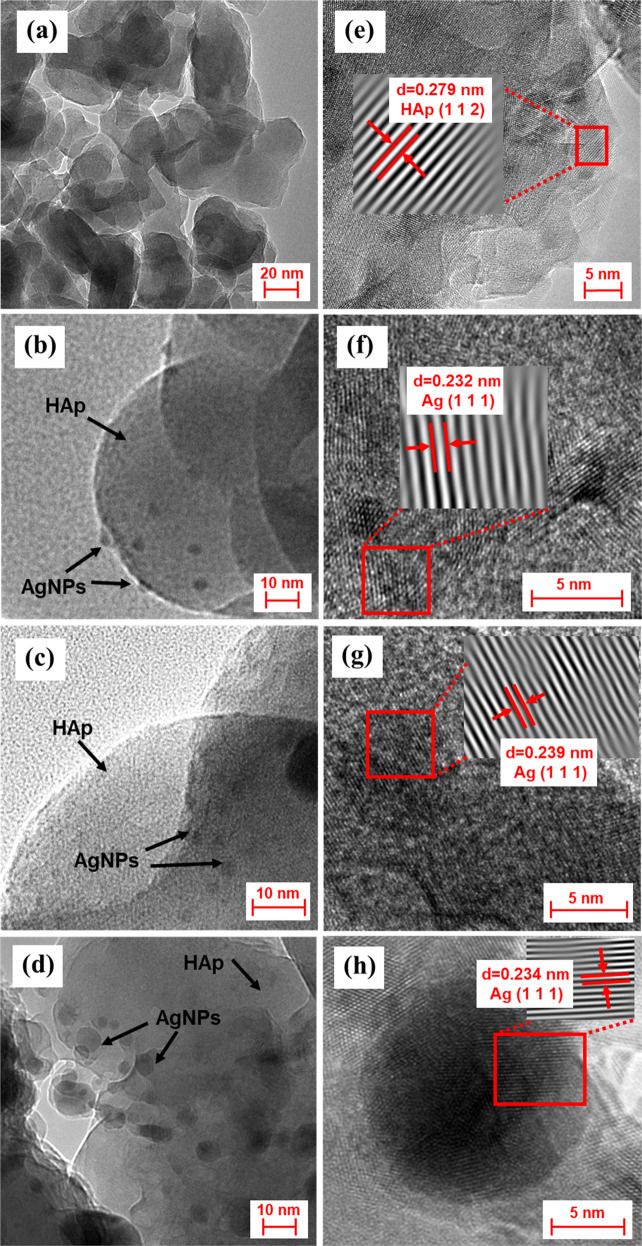
HRTEM and lattice fringe images of the prepared samples: **a**, **e** HAp, **b**, **f** HAp/AgNPs-1, **c**, **g** HAp/AgNPs-3, and **d**, **h** HAp/AgNPs-5 nanocomposites

Figure 5 presents the HRTEM images of HAp and HAp/AgNPs nanocomposite. By referring to Fig. 5a, sample prepared from the calcination of chicken bone exhibits irregular-like crystal structure. The lattice fringe with a interplanar spacing of 0.279 nm as observed in HRTEM micrograph taken at successively higher magnification (Fig. 5e) is corresponded to the (1 1 2) plane of HAp. As for the nanocomposite sample, decoration of spherical AgNPs onto the HAp surface can be evidenced by the formation of dark-colored spots deposited on the surfaces of HAp crystal. In addition, the lattice fringe spacings of 0.232, 0.239, and 0.234 nm as shown in Fig. 5f, g, h, respectively, are corresponded to (1 1 1) plane of fcc Ag. This further confirms the formation of AgNPs in the nanocomposite sample. Moreover, the amount of AgNPs adhered to the HAp surface increased when the AgNO_3_ concentration used increase. It can also be observed from Fig. 5d that the decorated AgNPs were the biggest when the AgNO_3_ concentration used is 5 wt.%.

### XPS analysis

**Fig. 6:**
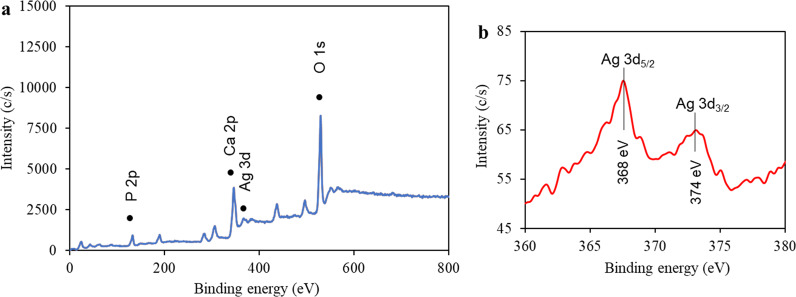
XPS spectra of HAp/AgNPs-5 nanocomposite: **a** XPS survey spectrum and **b** XPS high-resolution spectrum of the Ag 3d state

XPS analysis was employed to elucidate the chemical state of the constituents of HAp/AgNPs-5 nanocomposite as well as to further confirm the formation of AgNPs in the HAp. Figure 6a shows the full XPS survey scan of HAp/AgNPs-5 nanocomposite. The survey scan of HAp/AgNPs-5 nanocomposite corroborates the presence of P, Ca, Ag, and O elements through their respective binding energies at 132.72, 346.72, 366.72, and 529.72 eV. By referring to Fig. 6b, the high-resolution spectrum of Ag shows the characteristic 3d doublet peaks that are corresponding to the Ag 3d_5/2_ and Ag 3d_3/2_ of metallic Ag atom (Ag^0^ state), respectively. This result is comparable to the standard Ag 3d binding energies for metallic Ag as established by previous studies [39, 40]. This result indicates that the Ag^+^ precursor was reduced to metallic Ag with the aid of DMF and PVA as reducing and stabilization agents.

### Proposed mechanism for the formation of HAp/AgNPs nanocomposite

**Fig. 7:**
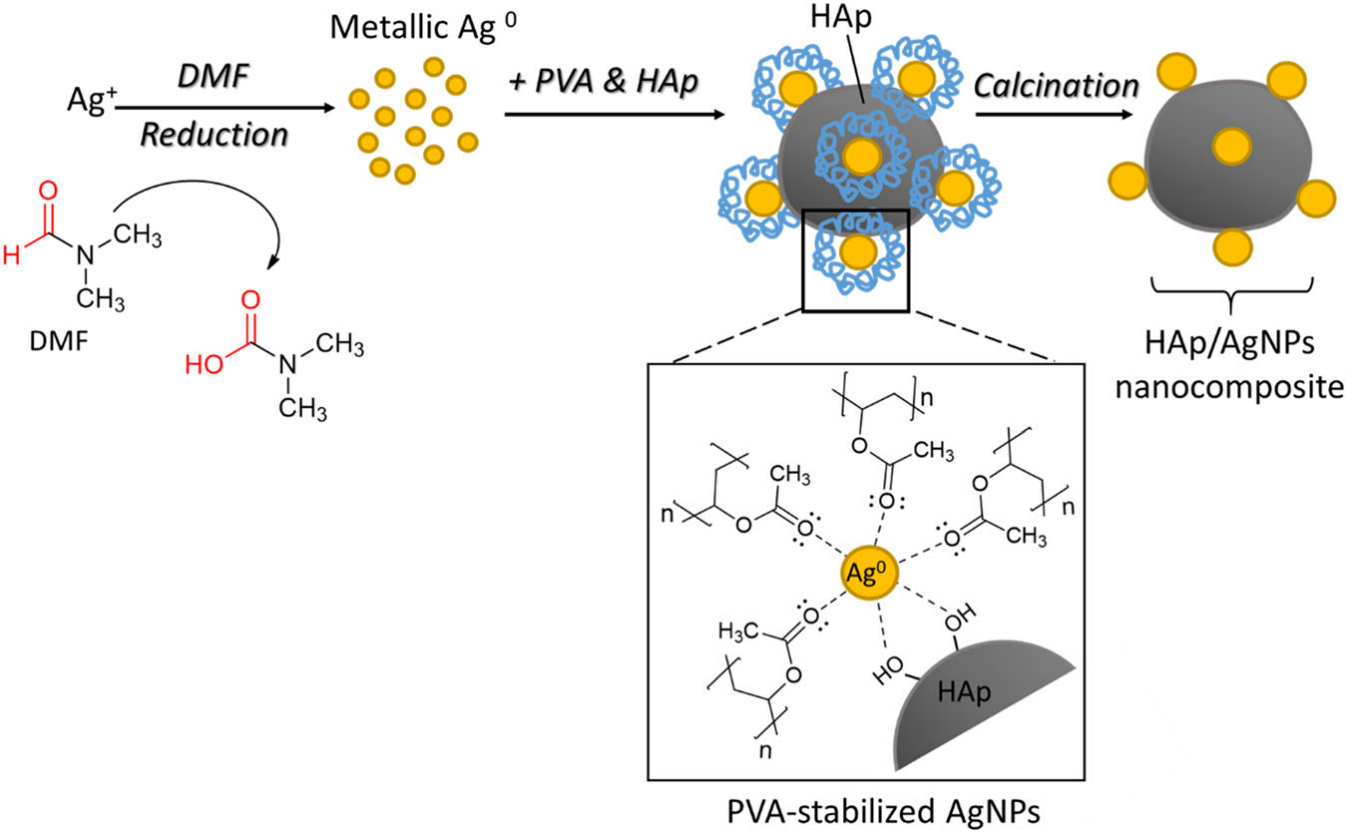
Proposed mechanism for the formation of HAp/AgNPs nanocomposite via chemical reduction route using DMF and PVA as reducing and capping agents, respectively

The proposed formation mechanism of HAp/AgNPs nanocomposites via chemical reduction of Ag salt precursor by DMF and stabilization offered by PVA is illustrated in Fig. 7. It is well known that the DMF can act as a powerful reducing agent for the reduction of noble metallic ions, while also serving as solvent [41]. When AgNO_3_ is added into DMF, the –C=O group of DMF can be easily oxidized to –COOH group in the presence of Ag^+^ ions of high oxidation power, which will concomitantly lead to the reduction of Ag(I) ions into metallic Ag(0) metal by donating electron during oxidation process [42].

Upon the addition of metallic Ag solution into the HAp/PVA suspension, PVA will serve as capping agent to hinder the excessive agglomeration of Ag particles in the reaction mixture during coalescence process. In this context, the AgNPs are likely to be stabilized through strong associations between AgNPs surfaces and oxygen atom in PVA, and a protecting covered layer would be generated on the surface of AgNPs. This layer will hamper the agglomeration of particles by imparting steric repulsive forces between AgNPs [43]. Meanwhile, HAp in the reaction mixture will also serve as a nucleating site for the adherent and deposition of AgNPs. This is because the electron-rich –OH groups found on the HAp surface present a high affinity for Ag atom that enables the adsorption and coordination of Ag atom via oxygen atom on the HAp. Upon completion of reaction, PVA can be thus removed from HAp/AgNPs nanocomposite sample by thermal treatment.

### Ag^+^ ions release study

**Fig. 8:**
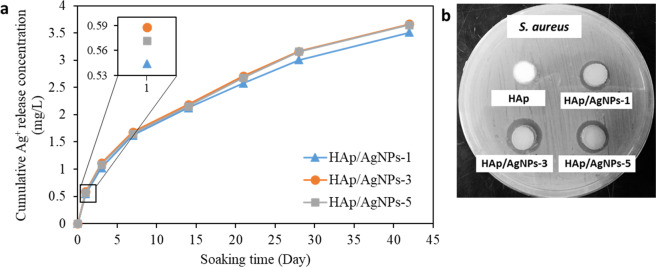
**a** The cumulative release concentration of Ag^+^ after soaking in PBS for different time, **b** antibacterial activity of all samples against *S. aureus* in the form of inhibition zone by agar well diffusion

The Ag^+^ ion release behavior of all HAp/AgNPs nanocomposite samples in PBS from 1 to 42 days is depicted in Fig. 8a. The obtained result shows that the Ag^+^ ions release in a sustain manner throughout the 42 days of immersion test. The sustain release behavior of Ag^+^ ion is beneficial for providing long-term antibacterial action during their application period. It is also noted that the Ag^+^ ion release concentration of all HAp/AgNPs nanocomposites is below the cytotoxicity level (10 mg/L) toward human cells, as presented by the previous literature study [34]. In overall, sample HAp/AgNPs-3 showed the highest Ag^+^ ion release concentration, which followed by HAp/AgNPs-5 and HAp/AgNPs-1 nanocomposite.

### Antibacterial activity

AgNPs have been prominent for its bactericidal properties with efficiency that increases as its nanoparticle size decreases [44]. Therefore, decoration of AgNPs onto the HAp surface is anticipated to impart antibacterial property to HAp. Figure 8b shows the antibacterial ability of all prepared samples formed using various AgNO_3_ concentration in the form of zone of inhibition. It can be observed that the unmodified pure HAp sample exhibited no zone of inhibition that was expected due to the lack of bactericidal properties of HAp in its pure form. By contrast, all the HAp/AgNPs nanocomposite samples showed a clear zone of inhibition surrounding the well, thus dictate their substantial antibacterial effect against *S. aureus*. The bactericidal activity of all the HAp/AgNPs nanocomposites was ascribed to the presence of AgNPs decorated on the HAp that may lead to the following antibacterial mechanisms to inactivate several critical physiological functions of bacterial cell: (a) interaction of the released Ag^+^ ions with the bacterial membrane that will alter and disrupt the membrane and causing cell death [45], (b) interaction of the released Ag^+^ ions with sulfur and phosphorus moieties of DNA to halt DNA replication, (c) production of reactive oxygen species that will damage the intracellular active components and lead to bacterial cell death [46, 47]. It is also discernible that the antibacterial efficiency of the nanocomposite increases as the AgNO_3_ concentration increases. This is ascribed to the presence of higher AgNPs content at increasing AgNO_3_ concentration (as evidenced by EDX analysis) that can liberate more Ag^+^ ions to perform inhibitory action against bacteria. It is noteworthy to mention that the inhibition zone exhibited by HAp/AgNPs-3 is much larger than in HAp/AgNPs-5 nanocomposite, which can be ascribed to the smaller size effect of the AgNPs that can release Ag^+^ ions faster to attain the highest antibacterial activity [48]. This result is consistent with the trend of the aforementioned Ag^+^ ions release profile. Notably, all HAp/AgNPs nanocomposites obtained in this research showed greater antibacterial activity (as indicated by the bigger inhibition zone) against *S. aureus* as compared to the one reported by the previous research [34], therefore indicate the potential of the proposed synthetic route for imparting antibacterial property.

### In vitro bioactivity study

**Fig. 9:**
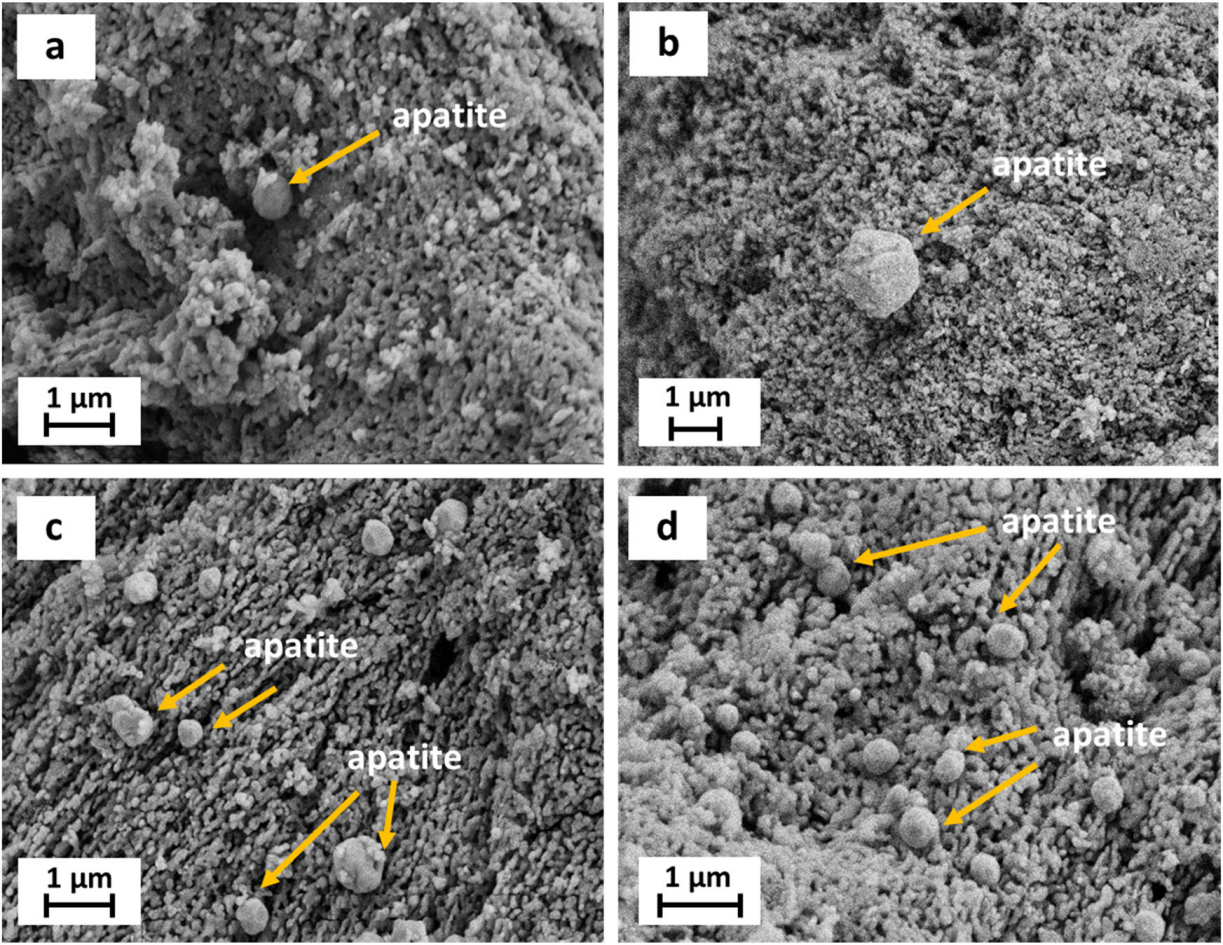
FESEM images of **a** HAp, **b** HAp/AgNPs-1, **c** HAp/AgNPs-3, and **d** HAp/AgNPs-5 nanocomposites after soaking in HBSS solution for 28 days

Formation of biologically active apatite is an indispensable prerequisite for direct bone-implant bonding, i.e., the bioactivity properties of a biomaterial [49]. In this study, the effect of chemical reduction treatment on the apatite formation ability of HAp in HBSS solution is identified by FESEM. As can be observed in Fig. 9a, spherically shaped apatite precipitates could be visualized in pure HAp sample after exposed to HBSS solution for 28 days. This proves the bioactive nature of the HAp that was derived from chicken bone wastes. After chemical reduction and calcination processes, the amount of apatite precipitated on the HAp surface increases proportionally with an increase of AgNO_3_ concentration used, thus demonstrating the impact of increasing AgNO_3_ concentration in enhancing the in vitro bioactivity of HAp. The enhancement in bioactivity via apatite formation can be related to the dissolution of HAp or HAp/AgNPs nanocomposite. In this study, it is assumed that some of the Ca^2+^ sites in the HAp lattice were substituted and occupied by Ag^+^ ions during chemical reduction reaction, which tends to disrupt the lattice structure of HAp and thus accelerating the dissolution rate of HAp in HBSS solution [50]. Therefore, the faster dissolution of soluble ions (i.e., Ca^2+^, P^4–^ ions) to the HBSS solution escalates the apatite formation and precipitation on HAp/AgNPs nanocomposite samples, especially in HAp/AgNPs-3 and HAp/AgNPs-5 nanocomposite samples.

## Conclusion

In this work, HAp/AgNPs nanocomposite with combined antibacterial and bioactivity characteristics had been successfully fabricated via chemical reduction and thermal calcination methods. Based on XRD, FESEM-EDX, and HRTEM results, the amount and size of AgNPs decorated on the surface of HAp particles are depended on the concentration of AgNO_3_ used. It was revealed that increasing AgNO_3_ concentration will increase the amount and size of the decorated AgNPs. Moreover, it was further shown that the chemical reduction and thermal calcination processes did not introduce any other phase or impurity to the final HAp/AgNPs nanocomposites. In the meantime, agar well diffusion test showed that all HAp/AgNPs nanocomposites exhibit effective bactericidal effect against *S. aureus* bacteria, where the antibacterial efficiency is relied on the amount and size of deposited AgNPs. When AgNO_3_ concentration used >1 wt.%, the bioactivity of the resulting nanocomposites displayed significant improvement as compared to pristine HAp. In overall, HAp/AgNPs-3 nanocomposite exhibits the optimal antibacterial and bioactivity properties that could be promising to be used as implant material for dental and orthopedic applications.
